# Body mass index and complications after obstetric anal sphincter injury, 8 weeks postpartum

**DOI:** 10.1007/s00192-022-05328-w

**Published:** 2022-09-09

**Authors:** Linda Hjertberg, Sofia Pihl, Marie Blomberg, Eva Uustal

**Affiliations:** 1 Department of Obstetrics and Gynecology in Norrköping, and Department of Biomedical and Clinical Sciences, Linköping University, Linköping, Sweden; 2 Department of Obstetrics and Gynecology in Linköping, and Department of Biomedical and Clinical Sciences, Linköping University, Linköping, Sweden

**Keywords:** Anal incontinence, Body mass index, Obstetric anal sphincter injury, Patient-reported outcome, Urinary incontinence

## Abstract

**Introduction and hypothesis:**

The impact of body mass index (BMI) on pelvic floor recovery after an obstetric anal sphincter injury (OASI) is unclear. The aim of this study was to evaluate the hypothesis that urinary incontinence (UI) and anal incontinence (AI) are more common in overweight and obese women than in normal-weight women 8 weeks postpartum in women with OASI.

**Methods:**

A population-based cohort study including 6,595 primiparous women, with an OASI, delivered between 2014 and 2019. Exposure and questionnaire data were retrieved from the Swedish Perineal Laceration Registry.

Uni- and multivariate analyses were used to compare normal-weight (BMI ≤24.9, reference), overweight (25.0–29.9), and obese (≥ 30) women with regard to UI and AI at 8 weeks post-partum.

**Results:**

Multivariate analyses showed an increased risk for urinary incontinence (OR 1.54, 95% CI 1.27–1.87) among overweight women as well as among obese women (OR 1.72, 95% CI 1.32–2.24). In contrast to our hypothesis, both overweight women (OR 0.68, 95% CI 0.56–0.83) and obese women (OR 0.65, 95% CI 0.49–0.87) were at a decreased risk for any gas and/or faecal incontinence after adjustment to possible confounding factors.

The absolute rate of AI was 40.1% among normal-weight women, 34.2% among overweight women, and 29.1% in the obese group.

**Conclusions:**

Urinary incontinence is more common, whereas AI is less common among overweight and obese women than in primiparous women with a BMI <24.9, 8 weeks after an OASI. The new finding, that overweight women report less AI than normal-weight women, merits further study.

**Supplementary information:**

The online version contains supplementary material available at 10.1007/s00192-022-05328-w

## Introduction

Obstetrical anal sphincter injury (OASI) is a major risk factor for anal incontinence (AI), defined as involuntary loss of feces or gas, fecal incontinence (FI), defined as involuntary loss of feces [1], urinary incontinence (UI), sexual dysfunction, and affected quality of life [2, 3]. The prevalence of OASI among primiparous women varies between 1.4% and 16%, with a large variation between different countries around the world [4]. OASI includes both third- and fourth-degree perineal lacerations, involving the anal sphincter muscle complex and/or the anorectal epithelium [5].

It is well known that maternal overweight and obesity are risk factors for several adverse obstetric outcomes and postpartum complications [6, 7]. However, two large cohort studies have shown that obese women have a decreased risk for OASI compared with normal-weight women. It is not known whether this is a true decreased risk or if it is due to insufficient diagnostics in this group [8, 9]. The prevalence of obesity and overweight is increasing among women of childbearing age globally. In Sweden in 2019, 27.2% of the pregnant women were overweight (body mass index [BMI] 25–29.9) and a further 15.7% were obese (BMI ≥30) [10].

The prevalence of AI and UI after an OASI varies in the literature, as do the definitions of AI and UI used in studies. Risk factors described for developing FI and UI after an OASI are age, race, antenatal UI, and BMI [10]. Another study indicates that maternal obesity is a risk factor for anal incontinence after childbirth regardless of whether an OASI had been diagnosed or not [11]. For women at risk of developing AI and UI after an OASI, it is of great importance to target prevention, treatment and follow-up related to these risks [12]. To the best of our knowledge, no previous population-based studies have examined the association between maternal BMI and AI, UI, and quality of life after a first-time vaginal delivery complicated by an OASI.

Thus, the primary aim of this study was to examine the impact of maternal BMI on pelvic floor dysfunction at 8 weeks after a first-time vaginal delivery complicated by an OASI.

The hypothesis was that AI and UI were more common in obese and overweight women at 8 weeks postpartum than in normal-weight women.

## Materials and methods

This is a nationwide population-based cohort study based on data from the Swedish Perineal Laceration Registry (PLR) between January 2014 and April 2019 including 6,595 women who had a first-time vaginal birth with an OASI.

The PLR is a section of the Swedish National Quality Register of Gynecological Surgery [13]. The register has previously been described in detail [14, 15]. Briefly, the PLR is a growing nationwide register that started in 2014, and as of 2021 has encompassed all delivery units in Sweden. The Swedish delivery units have joined the register successively since the PLR started.

The PLR provides survey data of patient-reported outcome and experience, as well as data extracted from women’s medical records. Patient-reported data are collected by postal or electronic questionnaires at three points after delivery: first, before discharge from hospital after childbirth concerning pre-pregnancy information, second, at 8 weeks, and finally at 1 year postpartum, concerning pelvic floor function and complications. For the purposes of the present study, the pre-pregnancy and 8-week questionnaires were analysed (Appendices 1 and 2). The questionnaires include questions about several aspects of pelvic floor dysfunction. In the target group interviews preceding the introduction of the PLR, questionnaire brevity was considered very important. Because of that, and to attain rates of completed questionnaires that are as high as possible, only a few selected questions about pelvic floor function were chosen. The questionnaires have been face-, context-, consistency- and content-validated. Those women who reported involuntary leakage of gas or feces were also presented with further questions that comprise the Wexner score for anal incontinence [3]. The Wexner scores of the BMI groups were compared, with a cut-off level ≥2 to define a degree of anal incontinence that affects quality of life after an OASI [3] .

Extracted maternal and obstetrical characteristics were age, BMI, prevalence of diabetes mellitus types I and II (DM), prevalence of inflammatory bowel disease (IBD, Crohn’s disease or ulcerative colitis), prevalence of urinary and anal incontinence, fetal presentation at birth, fetal birth weight, duration of second stage of labour, episiotomy at delivery and mode of vaginal delivery. Data related to the degree and repair of the perineal laceration was anaesthesia during repair and prophylactic antibiotics during repair, suture techniques, suture materials used for the external, internal sphincter and the perineum.

The study population was divided into five BMI groups: underweight (>18.5 kg/m^2^), normal weight (18.5–24.9 kg/m^2^), overweight (25.0–29.9 kg/m^2^), obesity (30–34.9 kg/m^2^) and morbid obesity (≥35 kg/m^2^) for analysis concerning maternal and obstetrical characteristics, as well as repair of the perineal laceration.

Outcomes of the present study, in relation to maternal BMI (three groups BMI ≤ 24.9, overweight [25.0–29.9 kg/m^2^], and obesity [≥30]) were: prevalence of urinary incontinence, defined as urinary incontinence once a week or more often; anal incontinence defined as any anal incontinence; urinary retention yes/no; any infections treated with antibiotics; wound-complications yes/no; re-operation yes/no; unplanned visits at an out-patient unit; and other complications as reported 8 weeks postpartum. The used definition of anal incontinence is reported involuntary loss of feces or flatus [1]. The Wexner score was calculated based on the supplementary questions only in women who answered “yes” to the question about incontinence of gas or feces at 8 weeks postpartum. The Wexner score contains questions about incontinence of gas, liquid stool, solid stool, use of pads, and change of lifestyle, and how often these parameters are present (never/rarely/1–3 times monthly/1–3 times weekly/daily).

For the purposes of dichotomization, women who reported symptoms once a week or more often were categorized into one group. Women who answered no to the question about any gas and/or fecal incontinence, and women with symptoms less frequent than once a week were categorised into a comparative group.

The total Wexner score was also dichotomised into the groups “Wexner score ≥2” and “Wexner score <2” (see Table 3).

### Ethical approval

The Regional Ethical Review Board in Linköping approved the study on 20 April 2016; Dnr 2016/144-31.

### Statistics

Data were analysed using SPSS version 27 (IBM, Armonk, NY, USA). The descriptive analyses of the categorical background data were presented as total number of patients (*N*), and percentage of each variable in each BMI group. Continuous data were presented as mean and one standard deviation (SD) or median and interquartile range (IQR), if not normally distributed. Pearson’s Chi-squared test was used to compare categorical variables. Analysis of variance was used to compare mean value of age.

*p* Values < 0.05 were considered significant. Variables with few available cases were not statistically analysed. Binary logistic regression analyses were used for comparison among the three BMI groups, normal weight (BMI ≤24.9), overweight (25.0–29.9) and obesity (≥30) with BMI ≤24.9 as the reference group. Risk estimates were presented as crude odds ratios (ORs) with 95% confidence intervals (CIs).

Multivariate analysis was used for comparison among the BMI groups. Potential confounders were selected by univariate analysis. Variables with a significant relation to the outcome in the primary multivariate analysis were included in the final adjusted odds ratio (OR) calculation. Risk estimates are presented as adjusted OR with 95% CI.

Non-parametric tests were used to analyse the duration of the second stage of labour, and days to resumption of normal ADL, in the numerical background data. Pearson’s Chi-squared test was used to analyse categorical parameters.

## Results

The study population consisted of 6,595 women with a first-time vaginal birth and who had a third- or fourth-degree perineal laceration registered in the Swedish PLR during the study period.

Background data are shown in Table 1. In the study population 3.1% of the women were underweight, 60.4% were normal weight, 25.2% were overweight and 11.3% were obese (8.1% obesity class I and 3.2% total in obesity classes II and III). There was no difference in BMI between responders and non-responders.

**Table 1:** Maternal and obstetrical characteristics in relation to maternal body mass index (BMI)

		BMI <18.5 (*N*=203)	BMI 18.5–24.9 (*N*=3,984)	BMI 25.0–29.9 (*N*=1,662)	BMI 30–34.9 (*N*=536)	≥35 (*N*=210)	*p* value
Age, years, mean (SD)		28.4 (4.75)	29.74 (4.45)	29.24 (4.67)	29.09 (4.66)	28.60 (4.54)	0.050
Diabetes Mellitus, *n* (%)	Yes	3 (1.5)	25 (0.6)	23 (1.4)	7 (1.3)	3 (1.4)	0.020
Inflammatory bowel disease, *n* (%)	Yes	3 (1.5)	37 (0.9)	9 (0.5)	1 (0.2)	2 (1.0)	0.127
Pre-pregnancy urinary incontinence, *n* (%)	Yes	1 (0.8)	30 (1.0)	20 (1.7)	6 (1.7)	9 (6.4)	<0.005
Pre-pregnancy gas and/or fecal incontinence, *n* (%)	Yes	4 (3.3)	146 (5.0)	42 (3.6)	13 (3.6)	9 (6.6)	<0.005
Duration of the second stage of labour, min, median (IQR)		43 (28–62)	40 (25–60)	38 (22–58)	33 (21–53)	29 (19–52)	<0.005
Fetal presentation at birth, *n* (%)	Occiput anterior	189 (93.1)	3,754 (94.2)	1,545 (93.0)	505 (94.2)	197 (93.8)	0.709
	Occiput posterior	12 (5.9)	176 (4.4)	91 (5.5)	24 (4.5)	7 (3.3)	
	Breech or foot	0	12 (0.3)	2 (0.1)	1 (0.2)	1 (0.5)	
	Other	2 (1.0)	38 (1.0)	22 (1.3)	6 (1.1)	3 (1.4)	
Episiotomy, *n* (%)	Yes	35 (17.2)	475 (11.9)	212 (12.8)	60 (11.2)	17 (8.1)	0.046
Mode of delivery, *n* (%)	Spontaneous vaginal	149 (73.4)	2,890 (72.5)	1,191 (71.7)	394 (73.5)	162 (77.1)	0.524
	Instrumental vaginal	54 (26.6)	1,094 (27.5)	471 (28.3)	142 (26.5)	48 (22.9)	
Fetal birth weight, mean g (SD)		3,514 (425.61)	3,664 (469.42)	3,721 (508.73)	3,755 (512.05)	3,786 (573.44)	0.050

Categorical data are presented as number and percentage. Continuous data are presented as mean and one standard deviation (SD) or median and interquartile range (IQR). Categorical characteristics were compared using a Pearson’s Chi-squared test. One-way analysis of variance was used for comparison of normally distributed continuous variables and Kruskal–Wallis test for not normally distributed continuous variables

The rate of episiotomy was lower with higher BMI (*p*= 0.046). There was a significant difference in the proportion of episiotomies between the underweight group and the morbidly obese group (*p*< 0.050). Also, the duration of the second stage of labour decreased with higher BMI (*p*<0.005). A statistically significant difference was seen in the fetal birth weight between the BMI groups (*p*<0.050). There were no other differences regarding obstetrical characteristics between the BMI groups. The ratio between the perineal lacerations (third or fourth degree) was similar within the respective BMI group.

Data related to the repair of the perineal laceration are shown in Table 2. The use of prophylactic antibiotics during repair, suture techniques and suture materials did not differ substantially among the BMI groups.

**Table 2:** Data related to the perineal laceration, degree and repair, in relation to maternal body mass index (BMI)

		BMI <18.5 (*N*= 203)	BMI 18.5–24.9 (*N*=3,984)	BMI 25–29.9 (*N*=1,662)	BMI 30–34.9 (*N*=536)	BMI ≥35 (*N*=210)	*p* value
Perineal laceration, *n* (%)	3rd degree	185 (91.1)	3,667 (92.0)	1,538 (92.5)	495 (92.4)	191 (91.0)	0.859
	4th degree	18 (8.9)	317 (8.0)	124 (7.5)	41 (7.6)	19 (9.0)	
Epidural anaesthesia during surgery, *n* (%)	Yes	38 (18.7)	716 (18.0)	335 (20.2)	100 (18.7)	35 (16.7)	0.385
Prophylactic antibiotics during suturing, *n* (%)	Yes	84 (41.4)	1,545 (38.8)	649 (39.0)	192 (35.8)	80 (38.1)	0.867
Suture technique external sphincter, 3rd degree, *n* (%)	End-to-end	137 (67.5)	2,966 (74.4)	1,250 (75.2)	405 (75.6)	151 (71.9)	0.061
	Overlap	31 (15.3)	388 (9.7)	166 (10.0)	50 (9.3)	27 (12.9)	
Suture technique external sphincter, 4th degree, *n* (%)	End-to-end	13 (6.4)	236 (5.9)	88 (5.3)	30 (5.6)	10 (4.8)	0.768
	Overlap	4 (2.0)	61 (1.5)	25 (1.5)	7 (1.3)	5 (2.4)	

Categorical data are presented as number and percentage

Categorical data were compared using a Pearson’s Chi-squared test

The outcomes of the 8-week follow-up questionnaires are presented in Table 3. Out of the study population, 75% (4,978/6,595) of the women with available data on BMI provided answers to the 8 week follow up questionnaires. There was no difference in BMI between responders and non-responders.

**Table 3:** Patient-reported complications after obstetric anal sphincter injury from the 8-week follow-up questionnaire

	BMI <24.9		BMI 25.0–29.9			BMI >30		
	*N* (%)	OR	*N* (%)	OR	95% CI	*N* (%)	OR	95% CI
Reported urinary incontinence	462/2,959 (15.6)	1.00	246/1,178 (20.9)	**1.43**	1.20–1.69	111/519 (21.4)	**1.47**	1.17–1.86
Reported urinary retention	43/3,148 (1.4)	1.00	20/1,263 (1.6)	1.16	0.68–1.98	5/547 (0.9)	0.67	0.26–1.69
Reported incontinence for gas or feces	1,258/3,141 (40.1)	1.00	431/1,260 (34.2)	**0.85**	0.75–0.97	159/547 (29.1)	**0.73**	0.60–0.88
Reported gas incontinence^a^	807/1,252 (64.5)	1.00	267/426 (62.7)	0.93	0.74–1.16	95/158 (60.1)	0.83	0.59–1.17
Reported incontinence for loose stool^a^	201/1,250 (16.1)	1.00	59/427 (13.8)	0.84	0.61–1.15	24/158 (15.2)	0.94	0.59–1.48
Reported incontinence for solid stool^a^	80/1,245 (6.4)	1.00	26/425 (6.1)	0.95	0.60–1.50	5/158 (3.2)	0.48	0.20–1.19
Reported usage of pads for fecal incontinence^a^	108/1,247 (8.7)	1.00	28/427 (6.6)	0.74	0.48–1.14	14/158 (8.9)	1.03	0.57–1.84
Change of lifestyle due to fecal incontinence^a^	314/1,247 (25.2)	1.00	108/416 (25.4)	1.01	0.78–1.30	36/158 (22.8)	0.88	0.59–1.30
Wexner score ≥2	111/3,097 (3.6)	1.00	33/1,220 (2.7)	0.75	0.50–1.11	18/525 (3.4)	0.96	0.58–1.59
Infections treated with antibiotics	203/2,902 (7.0)	1.00	84/1,173 (7.2)	0.88	0.50–1.53	32/506 (6.3)	1.64	0.83–3.24
Reported re-operation	177/3,148 (5.6)	1.00	65/1,263 (5.1)	1.00	0.73–1.36	28/547 (5.1)	0.99	0.64–1.52
Reported visit at outpatient unit	643/3,148 (20.4)	1.00	246/1,263 (19.5)	0.94	0.76–1.15	93/547 (17.0)	**1.36**	1.02–1.81
Reported wound complication, patient assessment	580/3,148 (18.4)	1.00	194/1,263 (15.4)	**0.80**	0.67–0.96	86/547 (15.7)	0.83	0.65–1.06
Reported complication, physician assessment	730/2,717 (26.9)	1.00	262/1,087 (24.1)	0.86	0.74–1.02	111/479 (23.2)	0.82	0.65–1.03

Not all questions were answered by all women and the number of replies and percentages is given in each row

Binary logistic regression analyses were used for comparison between the body mass index (BMI) groups

Risk estimates are presented as odds ratio (OR) with 95% confidence intervals (CIs)

Significant odds ratios in bold figures

^a^Questions included in the Wexner score system

Urinary incontinence was significantly more common among overweight and obese women than in normal-weight women (BMI ≤24.9). The absolute rate of urinary incontinence was 15.6% (*N*=462) among normal-weight women, 20.9% (*N*=246) among overweight women and 21.4% (*N*=111) in the obese group.

Overweight women with an OASI were at a 43% significantly increased risk for urinary incontinence (OR 1.43, 95% CI 1.20–1.69) compared with normal-weight women, the corresponding OR for obese women was 47% (OR 1.47, 95% CI 1.17–1.86). When adjusted for age and pre-pregnancy urinary incontinence, the risk for urinary incontinence remained significant, with an increased risk for urinary incontinence among both overweight women (OR 1.54, 95% CI 1.27–1.87) and obese women (OR 1.72, 95% CI 1.32–2.24; Table 4).

**Table 4:** Patient-reported complications after obstetric anal sphincter injury from the 8-week follow-up questionnaire

	BMI <24.9		BMI 25.0–29.9			BMI >30		
	*N* (%)	OR	*N* (%)	OR	95% CI	*N* (%)	OR	95% CI
Reported urinary incontinence*	462/2,959 (15.6)	1.00	246/1,178 (20.9)	**1.54**	1.27–1.87	111/519 (21.4)	**1.72**	1.32–2.24
Reported incontinence for gas or feces**	1,258/3,141 (40.1)	1.00	431/1,260 (34.2)	**0.68**	0.56–0.83	159/547 (29.1)	**0.65**	0.49–0.87
Reported wound complication patient assessment***	580/3148 (18.4)	1.00	194/1,263 (15.4)	**0.80**	0.67–0.95	86/547 (15.7)	0.84	0.65–1.07

Not all questions were answered by all women and the number of replies and percentages is given in each row

Potential confounders were selected by univariate analysis

Multivariate analyses were used for comparison among the body mass index (BMI) groups

Risk estimates are presented as adjusted odds ratio (OR) with 95% confidence intervals (CI)

Significant odds ratios in bold figures

The models presented are adjusted for the indicated significant variables *, **, ***

*Adjusted for age, and pre-pregnancy urine incontinence

**Adjusted for pre-pregnancy gas and/or feces incontinence, prophylactic antibiotic during repair, fetal birth weight and degree of perineal laceration (3rd and 4th degree)

***Adjusted for mode of delivery, and episiotomy

The risk for any anal incontinence was lower among overweight and obese women than in women with BMI ≤24.9. The absolute rate of reported incontinence for gas or feces was 40.1% (*N*=1,258) among normal weight women, 34.2% (*N*=431) among overweight women and 29.1% (*N*=159) in the obese group. Women who were overweight had a 15% decreased risk for incontinence of gas or feces (OR 0.85, 95% CI 0.75–0.97), whereas obese women had a 27% decreased risk, compared with normal-weight women (OR 0.73, 95% CI 0.60–0.88).

Adjustments were made for pre-pregnancy gas and/or fecal incontinence, fetal birth weight, degree of perineal laceration (3rd or 4th degree) and use of prophylactic antibiotics during repair. After adjustments, overweight women were at a decreased risk for any gas and/or fecal incontinence (OR 0.68, 95% CI 0.56–0.83) and obese women were at a decreased risk (OR 0.65, 95% CI 0.49–0.87).

Among the women reporting any incontinence for gas or feces, the type or extent of incontinence according to the Wexner score was not influenced by BMI. A cut-off level at a Wexner score of ≥2 was used to estimate the effect on quality of life in women with AI postpartum [3]. There was no difference in the reported quality of life between the BMI groups.

Overweight women were at a 20% decreased risk of wound complications compared with normal-weight women (OR 0.80, 95% CI 0.67–0.96). After adjustments were made for age, mode of delivery and episiotomy, the decreased risk for patient-reported wound complications remained significant (OR 0.80, 95% CI 0.67–0.95).

The rates of reported infections treated with antibiotics and reported re-operations did not differ over the BMI strata. Median number of days reported to resumption of normal ADL was 10 days among normal-weight women and overweight women, whereas obese women had a median time of return to resumption of normal ADL of 11 days.

The women’s report at 8 weeks regarding any complications was classified by the physician who had repaired the perineal laceration as no, mild or severe complications. The rate of complications was somewhat lower among overweight and obese women than among normal-weight women, but the association was not statistically significant.

## Discussion

This study showed that women who were obese or overweight were at a lower risk for developing AI at 8 weeks postpartum, compared with normal-weight women, after a first-time vaginal delivery complicated by an OASI. On the contrary, overweight and obese women were at a higher risk for developing UI at 8 weeks postpartum after a first-time vaginal delivery complicated by an OASI, compared with normal weight women. The result that overweight and obese women are at a lower risk of developing AI 8 weeks after a first-time vaginal delivery complicated by an OASI is a new finding. UI is well known to correlate with higher BMI in older age groups, even after deliveries without OASI [16]. That UI correlates with BMI as early as 8 weeks after OASI has not been shown before.

The results in the literature vary concerning the prevalence and risk factors of AI and UI after an OASI among primiparous women [17–19]. Different follow-up questionnaires, follow-up periods and definitions of the incontinence can account for these differences [1, 4]. A few previous studies have reported BMI as a risk factor of developing AI and UI after OASI. Contrary to our study results, a Danish study by Gommesen et al. showed that the risk for developing AI after a first-time delivery with an OASI increased with higher BMI. In their prospective cohort study including 200 primiparous women with an OASI, they found 1 year postpartum that the risk for AI increased by 8% per 1-unit increase in BMI [20]. Further, Burgio et al. investigated risk factors for fecal and urinary incontinence in primiparous women after an OASI [10]. Their study population was interviewed directly postpartum, at 6 weeks after delivery and after 6 months concerning UI and FI. Their study population consisted of 335 primiparous women with an OASI who completed interviews at 6 months postpartum. They used similar questions, regarding pre-delivery symptoms and postpartum symptoms of UI and AI, to those used in the present study. They found that FI was associated with higher pre-delivery BMI as well as race, antenatal UI and older maternal age at delivery. Their findings differ from our study results according to the risk for AI after a first-time vaginal delivery complicated by an OASI. Regarding the risk for UI after an OASI, the results concur. AI is less common than UI in general. Our considerably larger study population with 4,978 women included may explain that our results differ from those of other studies, namely a decreased risk for AI with a higher BMI.

Possibly, what seems to protect overweight and obese women from having an OASI in the first place [8, 9] could also protect them from AI in the aftermath. Women with overweight and obesity have a shorter second stage of labour than normal-weight women [21]. This could have a protective effect on levator muscle injury and the pudendal nerve. Also, overweight and obese nulliparous women have a higher anovaginal distance (AVD) in the active phase of labour. A higher AVD may play a protective role in the anovaginal complex [22], as well as for the levator ani muscle insertions and the pudendal nerve. These possible explanations merit further studies.

The higher risk for UI after a first-time vaginal delivery complicated by an OASI among overweight and obese women is less surprising as obese women have a higher intra-abdominal pressure [23]. Also, overweight and obesity increase the load on the pelvic floor [24]. This could contribute to the higher rates of UI in women who are overweight or obese than in normal-weight women after an OASI.

Wound infections in the postpartum period after having an OASI can cause wound dehiscence [25]. A prospective Danish cohort study including 200 primiparous women with an OASI, found that obese women were at a higher risk for wound infections and wound dehiscence than normal-weight women [26]. We did not find any increased risk for wound infection or wound complications in the higher BMI groups compared with normal-weight women. On the contrary, the overweight group were at a decreased risk for wound complications compared with normal-weight women. This could also contribute to a lower risk for developing AI in this BMI group. The obese group were at a 17% decreased risk for wound complications compared with normal-weight women.

At the 8-week follow-up questionnaire 458 women out of a total of 1,373 women reported changes in life-style due to fecal incontinence in the Wexner questionnaire. There was no difference among the BMI groups regarding the effect of AI on quality of life. Later on in life, both AI and UI have been shown to have a negative effect on the quality of life, regardless of BMI [27].

In summary, the present study highlights pelvic floor complications after OASI in overweight and obese women. These results lead to further questions regarding whether targeted interventions could improve pelvic floor function in this group of women.

A study by Mathé et al suggests that early pelvic floor muscle training (PFMT), starting within 1 month postpartum, after having an OASI, could reduce symptoms of AI and may prevent the medium-term consequences of AI [28].

The efficacy of PFMT postpartum in women who are obese or overweight compared with normal-weight women after OASI is not well described. A Cochrane review from 2020 implies that focus on the impact of BMI on PFMT in women with AI and UI postpartum should be further investigated [12].

A strength of the present study is that the PLR is a population-based register that contains a large cohort available for evaluation. The large number of primiparous women with OASI registered in the PLR makes it possible to analyse subgroups within the population.

Another advantage of this study is that the population in the PLR is gathered from several centres and the findings could be generalisable to settings with similar demographic profiles and delivery practices.

Limitations of register studies are generally incomplete coverage and quality issues in recorded data, such as missing values. The PLR was launched nationally in 2014 and participation has been voluntary for delivery wards. National coverage has increased yearly and as of 2021, all delivery wards have been using the PLR.

The criteria for maternal and obstetrical characteristics may vary to across the country, given the large study population and the high number of health care units involved. However, there is no reason to believe that these variations are impacted by maternal BMI.

## Conclusions

This large population-based study showed that women with higher BMI have more UI but less AI 8 weeks after a first-time vaginal birth complicated by an OASI, than women with normal BMI. These are new findings concerning PFD in the immediate postpartum period. The reason for the discrepancy between UI and AI, regarding possible preventive factors associated with a higher BMI, merits further study.

## Supplementary information

Appendix 1 Pre-pregnancy questionnaire. https://www.gynop.se/wp-content/uploads/2019/01/Health-prior-to-pregnancy-.pdf. Accessed May 10 2022 (PDF 201 kb)

Appendix 2 Eight-week questionnaire. https://www.gynop.se/wp-content/uploads/2019/01/Health-prior-to-pregnancy-.pdf. Accessed 10 May 2022 (PDF 125 kb)
