# Supplementary material for: Body mass index and complications after obstetric anal sphincter injury, 8 weeks postpartum
Source: Int Urogynecol J. 2022 Sep 9;33(12):3465–72. doi: 10.1007/s00192-022-05328-w (PMC9666295; doi:10.1007/s00192-022-05328-w)
Supplement: Supplementary file 1 — Pre-pregnancy questionnaire. https://www.gynop.se/wp-content/uploads/2019/01/Health-prior-to-pregnancy-.pdf. Accessed May 10 2022 (PDF 201 kb) [file 192_2022_5328_MOESM1_ESM.pdf]

Information about your health prior to pregnancy

There are gaps in the numbering of questions since certain questions are not relevant for you.

1. Date when the questionnaire is completed: ................-.........-........

9a. Before the pregnancy, did you have a feeling that something is bulging out from the vagina?

☐ Never ☐ Almost never ☐ 1–3 times per month ☐ 1–3 times per week ☐ Daily

10d. Did you experience urinary leakage or involuntary urination?

☐ Never ☐ Almost never ☐ 1–3 times per month ☐ 1–3 times per week ☐ Daily

If you answered Never – Almost never to question 10d above, go to question 12

Mark an X next to **one of the options** for each question 11b – 11d

11b. How often did you experience leakage of urine associated with physical activity, or when you laugh, cough or sneeze?

☐ Never
☐ 1–4 times per month
☐ 1–6 times per week
☐ Once a day
☐ More than once a day

11d. How often did you experience a sudden onset of a strong need to urinate, and leaked urine before you reached the toilet?

☐ Never
☐ 1–4 times per month
☐ 1–6 times per week
☐ Once a day
☐ More than once a day

12a. Before the pregnancy, you ever have problems emptying your bowels?

☐ Never ☐ Almost never ☐ 1–3 times per month ☐ 1–3 times per week ☐ Daily

12b. Before the pregnancy, did you ever have to push against the back wall of the vagina to empty your bowels?

☐ Never ☐ Almost never ☐ 1–3 times per month ☐ 1–3 times per week ☐ Daily

DO NOT COPY

13a. Did you have problems holding in stool or gas?

- ☐ No
- ☐ Yes

If you answered No to the above question, skip to question 14a

13b. Did you ever pass gas even when it is inappropriate?

- ☐ Never
- ☐ Almost never
- ☐ Yes, 1-3 times a month
- ☐ Yes, 1-3 times a week
- ☐ Yes, daily

13c. Did you experience leakage of loose stool?

- ☐ Never
- ☐ Almost never
- ☐ Yes, 1-3 times a month
- ☐ Yes, 1-3 times a week
- ☐ Yes, daily

13d. Did you experience leakage of firm stool?

- ☐ Never
- ☐ Almost never
- ☐ Yes, 1-3 times a month
- ☐ Yes, 1-3 times a week
- ☐ Yes, daily

13e. Did you use sanitary pads/protection because of stool leakage?

- ☐ Never
- ☐ Almost never
- ☐ Yes, 1-3 times a month
- ☐ Yes, 1-3 times a week
- ☐ Yes, daily

13f. Did your leakage problem affect your lifestyle?

- ☐ Never
- ☐ Almost never
- ☐ Yes, 1-3 times a month
- ☐ Yes, 1-3 times a week
- ☐ Yes, daily

DO NOT COPY

Questions about stomach problems:

14a. During the last three months before your pregnancy, did you have pain or discomfort in your abdomen?

- ☐ No
- ☐ Yes

14b. Was this abdominal discomfort/pain only present during menstruation or pregnancy?

- ☐ No
- ☐ Yes

If you only had pain during menstruation or pregnancy, continue to question 15a

When answering the questions below, think about what it was like the last three months before you became pregnant.

14c. During times of abdominal discomfort/pain**,** did the number of stools/bowel openings increase?

- ☐ Never or seldom
- ☐ Sometimes
- ☐ Often
- ☐ Most of the time
- ☐ Always

14d.During times of abdominal discomfort/pain, did the number of stools/bowel openings decrease?

- ☐ Never or seldom
- ☐ Sometimes
- ☐ Often
- ☐ Most of the time
- ☐ Always

14e. During times of abdominal discomfort/pain, did you tend to have looser stools?

- ☐ Never or seldom
- ☐ Sometimes
- ☐ Often
- ☐ Most of the time
- ☐ Always

DO NOT COPY

14f. During the abdominal discomfort/pain, did you tend to have harder stools?

- ☐ Never or seldom
- ☐ Sometimes
- ☐ Often
- ☐ Most of the time
- ☐ Always

14g. During the abdominal discomfort/pain, how often did you have loose stool?

- ☐ Never or seldom
- ☐ Sometimes
- ☐ Often
- ☐ Most of the time
- ☐ Always

14h. During the abdominal discomfort/pain, how often did you have hard stool?

- ☐ Never or seldom
- ☐ Sometimes
- ☐ Often
- ☐ Most of the time
- ☐ Always

14i. Did your bowel symptoms start with having had a “stomach bug” (a gastrointestinal infection)?

- ☐ No
- ☐ Yes

If you frequently experience the problems described in the questions above, you may have IBS, which causes intestinal problems that are not dangerous but that may be difficult at times.

If you would like more information, go to 1177.se and search for IBS or talk to your family doctor.

DO NOT COPY

15a. Did you have discomfort in the genital area during sexual intercourse in the last 3 months before your pregnancy?

☐ Yes ☐ No ☐ Not applicable ☐ Prefer not to answer

15b. If yes on question 15a, did you experience pain in the genital area during intercourse?

☐ No, no pain

☐ Yes, mild pain

☐ Yes, moderate pain

☐ Yes, severe pain

☐ Yes, unbearable pain

15d. If yes on question 15a, did you feel

that your vaginal opening is too small/narrow?	☐ Yes ☐ No
that your vaginal opening is too large/open?	☐ Yes ☐ No
pain in the vaginal opening?	☐ Yes ☐ No
other symptoms from the vaginal opening?	☐ Yes ☐ No

If yes, what type of symptoms? ……………………………………………………………

……………………………………………………………………………………

Have you had any problems understanding any question(s) in the questionnaire? ☐ No ☐ Yes
If Yes, write the number of the question and describe the problem:

....

....

....

....

46 Is there anything else that you consider to be important that we should know?

....

....

....

....

Name (of the person who filled in the questionnaire)

DO NOT COPY
