# Supplementary material for: Body mass index and complications after obstetric anal sphincter injury, 8 weeks postpartum
Source: Int Urogynecol J. 2022 Sep 9;33(12):3465–72. doi: 10.1007/s00192-022-05328-w (PMC9666295; doi:10.1007/s00192-022-05328-w)
Supplement: Supplementary file 2 — Eight-week questionnaire. https://www.gynop.se/wp-content/uploads/2019/01/Health-prior-to-pregnancy-.pdf. Accessed 10 May 2022 (PDF 125 kb) [file 192_2022_5328_MOESM2_ESM.pdf]

Your assessment after the repair of the perineal tear

(about 8 weeks)

There are gaps in the numbering of questions since certain questions are not relevant for you.

1. Date when the questionnaire is completed: ................-.........-........

2. How do you feel about the length of your hospital stay?

☐ Just right
☐ Too long
☐ Too short

3. Did you need to take pain relieving medicine because of your surgery, after you left the hospital?

☐ No
☐ Yes How many days? …….

5d. Do you experience urinary leakage or involuntary urination?

☐ Never ☐ Almost never ☐ Usually one time ☐ Usually two times ☐ More than two times

During the first time after delivery it is common with small amounts of urinary leakage. It usually resolves with time. If the problems persist, contact your midwife of district nurse for advice. There is treatment.

7. How many days after delivery did you need before you could carry out normal activities of daily living and manage **on your own** without more help than before the operation (e.g. personal hygiene, cooking food for yourself, making the bed, taking short walks)? ....days.

After childbirth almost everyone experiences problems to a greater or lesser degree from the genital area and the abdomen, but these problems resolve on their own.

It is therefore difficult to know what is normal after a tear during childbirth and what is a complication. Here is one way to think about the questions. If your problems have been severe enough to require prescription medicine or an extra recheck, the complication is usually considered to be *mild*.

If your problems have a long-term impact on your quality of life and/or result in the need to be re-operated, the complication is usually considered to be *serious*.

9. Have you had unexpected discomfort or complications due to the perineal tear?

☐ No, **skip to question 16a**
☐ Yes, mild
☐ Yes, severe/serious
☐ Yes, mild and severe/serious

DO NOT COPY

11a. Did you need to seek medical care during the period after surgery because of these problems/complications?

☐ No ☐ Yes

**If yes**, what medical facility did you visit?	Name of department and hospital/primary care centre that you visited
☐ The department where you had surgery<br><br>☐ Primary care centre<br><br>☐ Other medical facility	....<br><br>...

11b. If yes, when was the first time you visited the medical facility after you returned home?

☐ Within 1 week ☐ 1 to 2 weeks ☐ 2 to 4 weeks ☐ Later than 4 weeks

11c. If you answered yes to question 11a: Were you hospitalised because of the complication?

☐ No, I left the hospital the same day
☐ Yes, stayed for one night.
☐ Yes, stayed for two or more nights

11d. If you answered yes to question 11a: Did treatment of the complication include surgery?

☐ No ☐ Yes

11e. If you answered yes to question 11a: Were your problems/complications treated? Select one or more responses

☐ No
☐ Yes, monitored with additional recheck and/or investigation
☐ Yes, pain relieving medicine
☐ Yes, the wound dressing was changed
☐ Yes, other treatment. Describe:
....

....

Describe your problems/complications by choosing one or more of the following options.

12a. Tick the affected organ/parts of the body:

☐ Surgical wound ☐ Urinary bladder

☐ Blood vessels ☐ Urethra

☐ Uterus ☐ Vagina

☐ Nerve/Sensation ☐ Intestines

☐ Other (Describe in question 13f)

DO NOT COPY

12b. Did the complication result in any of the following problems:

☐ Rupture of the surgical wound to the abdominal cavity that required a new operation (wound dehiscence)

☐ Abnormal connection to the vagina, intestine or urinary bladder (fistula)

☐ None of the above problems

Different types of complications.

13a. Bleeding:

☐ Blood collection(hematoma) in the genital area

☐ Other bleeding (Describe in question 13f)

☐ No bleeding

13b. Infection:

☐ Urinary tract infection

☐ Vaginal infection, foul-smelling discharge

☐ Infection of surgical wound

☐ Uterine infection

☐ General blood poisoning (sepsis)

☐ Other infection (Describe in question

☐ No infection

If you ticked any option for infection, was the infection treated with an antibiotic/penicillin?

☐ No

☐ Yes

13c. Pain:

☐ Genital pain

☐ Pain located elsewhere (Describe in question 13f)

☐ No pain

13d. Urination problems:

☐ Difficulties emptying the bladder that required treatment

☐ Pain on urination persisting more than 1 month after surgery

☐ Other (Describe in question 13f)

☐ No urination problems

13e. General medical complication:

☐ Abnormal fatigue, lethargy

☐ Depression

☐ Severe constipation

☐ Blood clot (thrombosis, emboli)

☐ Other complication (Describe in question 13f)

☐ None of the above

with regular monitoring of blood thinners

DO NOT COPY

13f. Describe the problems/complications you ticked above:

....

....

14. Do you still have problems with anything that you specified in questions 9-13?

- ☐ No
- ☐ Yes

If yes, describe: ....

....

....

15. If you answered yes to any of questions 9-14, would you consent to allow us to read the relevant medical records before your surgery?

- ☐ No
- ☐ Yes

16a. Do you have problems holding in stool or gas?

- ☐ No
- ☐ Yes

If you answered no to the above question, skip to question <u>17a</u>

16b. Do you ever pass gas even when it is inappropriate?

- ☐ Never
- ☐ Almost never
- ☐ Yes, 1-3 times a month
- ☐ Yes, 1-3 times a week
- ☐ Yes, daily

16c. Do you experience leakage of loose stool?

- ☐ Never
- ☐ Almost never
- ☐ Yes, 1-3 times a month
- ☐ Yes, 1-3 times a week
- ☐ Yes, daily

DO NOT COPY

16d. Do you experience leakage of firm stool?

- ☐ Never
- ☐ Almost never
- ☐ Yes, 1-3 times a month
- ☐ Yes, 1-3 times a week
- ☐ Yes, daily

16e. Do you use sanitary pads/protection because of stool leakage?

- ☐ Never
- ☐ Almost never
- ☐ Yes, 1-3 times a month
- ☐ Yes, 1-3 times a week
- ☐ Yes, daily

16f. Does your leakage problem affect your lifestyle?

- ☐ Never
- ☐ Almost never
- ☐ Yes, 1-3 times a month
- ☐ Yes, 1-3 times a week
- ☐ Yes, daily

17. Do you have any additional problems related to the perineal tear?

- ☐ No
- ☐ Yes

Describe the problem(s):..

....

....

....

18a. Have you had, or are you planned for, any follow up after the delivery?

- ☐ Yes
- ☐ No
- ☐ Don’t know

DO NOT COPY

18b. If No or Don't know, do you need someone from the Department of Obstetrics and Gynaecology to contact you?

☐ No, I will get in touch if any problems arise.
☐ Yes, I would like someone to contact me regarding ……………………………………………

19. Have you had any problems understanding any question(s) in the questionnaire? ☐ No ☐ Yes

If Yes, write the number of the question and describe the problem:

....

....

....

....

....

Name (of the person who filled in the questionnaire)

DO NOT COPY
